# Autoreactive Antibodies Associated with Castleman Disease Triad

**DOI:** 10.1155/2024/9382107

**Published:** 2024-03-06

**Authors:** Jacqueline A. Turner, Ali Hakimi, Hannah Lee, Jeffrey T. Schowinsky, Jeffrey M. Sippel, Bradford J. Siegele, Raul M. Torres, William A. Robinson

**Affiliations:** ^1^Division of Medical Oncology, University of Colorado Anschutz School of Medicine, Anschutz Medical Campus, Aurora, CO, USA; ^2^Department of Immunology and Microbiology, University of Colorado School of Medicine, Aurora, CO, USA; ^3^University of Colorado Anschutz School of Medicine, Anschutz Medical Campus, Aurora, CO, USA; ^4^Division of Hematology and Oncology, Olive View-University of California Los Angeles Medical Center, Sylmar, CA, USA; ^5^Department of Pathology, University of Colorado School of Medicine, Aurora, CO, USA; ^6^Division of Pulmonology, University of Colorado Medicine, University of Colorado School of Medicine, Aurora, CO, USA

## Abstract

The Castleman triad has been described in a select few patients presenting with a retroperitoneal mass, mucocutaneous pemphigus vulgaris, and bronchiolitis obliterans. Here, we describe the Castleman triad in a 19-year-old male with unicentric hyaline vascular type Castleman disease (HV-CD). This patient presented with an array of positive antibodies, including anti-cyclic citrullinated peptide, anti-double-stranded DNA, and Sjogren's IgG. Interestingly, the patient's rheumatologic symptoms resolved after tumor resection, while his antibody profile remained relatively unchanged. HV-CD, with a triad presentation, was thought to be from a paraneoplastic syndrome secondary to an underlying lymphoproliferative disorder. The findings presented here identify multiple autoantibodies potentially contributing to this patient's presentation with HV-CD.

## 1. Introduction

Castleman disease (CD) is a rare, heterogeneous group of lymphoproliferative disorders with angiofollicular lymphatic hyperplasia, although, little is understood about its pathogenesis. CD may present with local (unicentric) or disseminated (multicentric) disease. The CD is further stratified microscopically as hyaline vascular, plasma cell, or mixed [1]. Due to the rare incidence of CD, few studies have systematically evaluated treatments and outcomes. There are few reports in the literature of CD presenting with rheumatologic findings, paraneoplastic pemphigus (PNP), and transient autoantibodies in the serum [2–4]. Treatment of unicentric CD (UCD) is surgical resection or rituximab if unresectable [5–7]. More recently, CD has been reported to be responsive to several immunotherapies (siltuximab, rituximab, tocilizumab) and antiviral drugs (ganciclovir, valganciclovir, and foscarnet for human herpes virus-8, HHV8, infected patients) [1, 8]. Yet, the immunopathophysiology and long-term outcomes of UCD remain understudied. We present a 19-year-old patient with the Castleman triad of retroperitoneal mass, mucocutaneous pemphigus vulgaris, bronchiolitis obliterans, and numerous *persistently* positive autoantibodies.

## 2. Case Presentation

**Figure 1:**
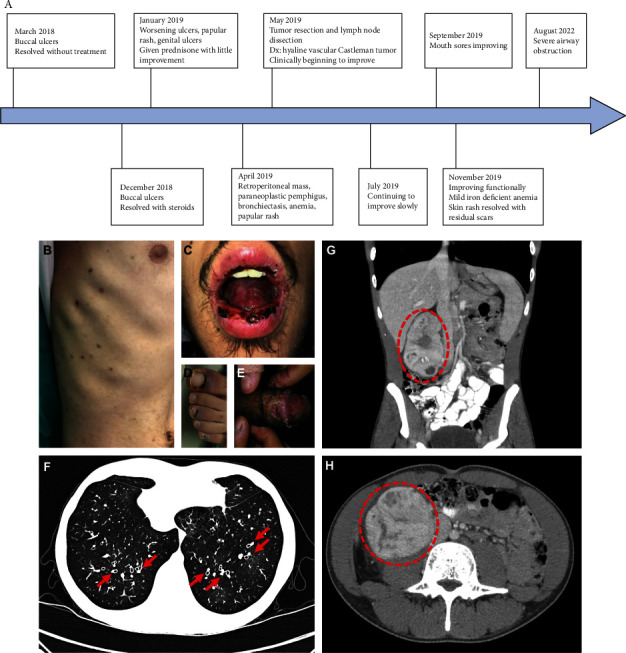
Clinical course and presentation of Castleman disease with retroperitoneal mass: (A) course of patient diagnosis and treatment. (B)–(E) physical findings from April 2019 showing (B) papular rash on the patient's trunk, (C) ulcerative stomatitis, (D) violaceous sanguineous cuticle encrustations, and (E) genital ulcers; (F) chest CT with red arrows indicating signet rings; (G, H) abdominal CT with red dotted circles indicating the retroperitoneal tumor.

A previously healthy 19-year-old male presented to the clinic with stomatitis in March 2018 (Figure 1(A)). His buccal biopsy revealed lichenoid interface dermatitis, which resolved without treatment. In December 2018, he presented again with buccal ulcers responsive to steroidal treatment. In January 2019, he returned with severe buccal ulcers, a papular rash along his trunk, painful genital ulcers, and blurry vision. Prednisone provided minimal relief. Three months later, he returned with worsening symptoms with new cuticle sanguineous crusting on his distal extremities, weight loss, pleuritic chest pain, arthralgias, and dyspnea with minimal exertion. The physical findings were consistent with mucocutaneous pemphigus vulgaris (Figure 1(B)–1(E)). Infectious workup for viruses, including HHV8, HIV, and other viremia and bacteremia, was unrevealing. Serologic testing for autoimmune antibodies was positive for several autoantibodies, including anti-nuclear antibodies, anti-cyclic citrullinated peptide, anti-double-stranded DNA, and Sjogren's IgG (Table 1). IgM and IgG against Mycoplasma pneumoniae and IgG against Chlamydia pneumoniae were positive, while the PCR detection of Mycoplasma pneumoniae was negative. CT of the chest, abdomen, and pelvis revealed ground glass opacities and bronchiectasis with signet rings (Figure 1(F)) and a 12 × 7.2 cm retroperitoneal mass with associated lymphadenopathy (Figures 1(G) and 1(H)).

**Figure 2:**
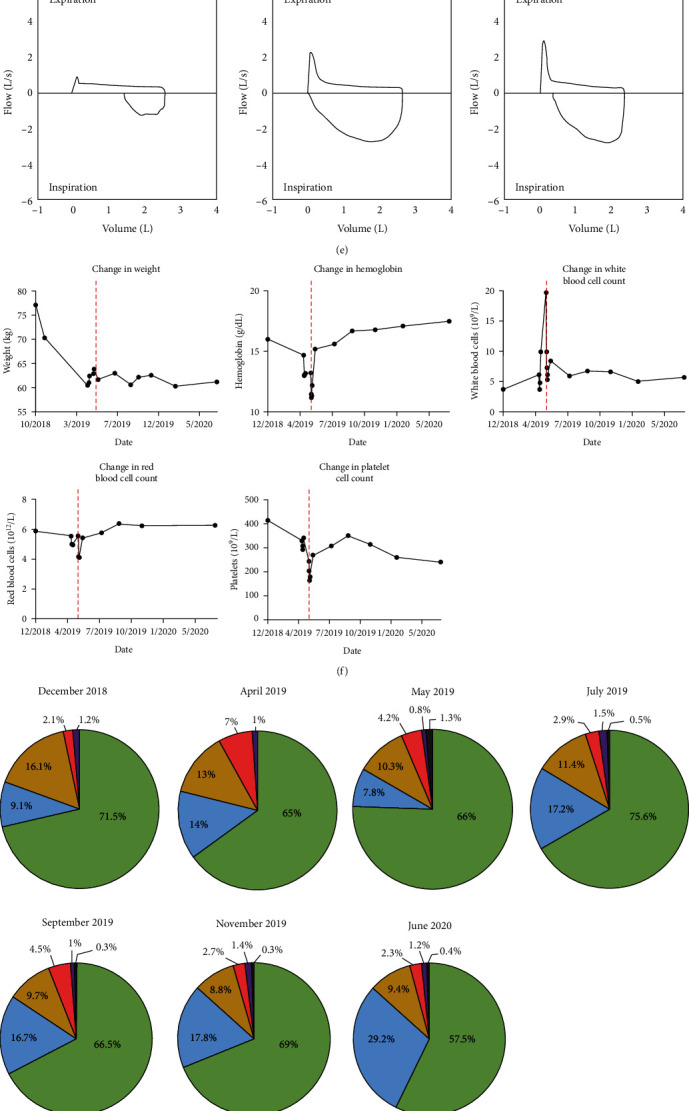
Diagnosis of unicentric hyaline vascular type Castleman disease: (a)–(d) immunohistochemical staining of the retroperitoneal mass showing (a) onionin-skinning and CD21 positive lymphocytes, (b) paracortical vascular penetration, (c) pan-IgG staining, and (d) flow cytometric immunophenotyping tumor biopsy and resection; (e) patient pulmonary function tests; (f) clinical data collected before and after surgery including weight, hemoglobin, white blood cell count, red blood cell count, and platelet count. Vertical dashed red line indicates the date of surgery; (g, h) white blood cell differential by (g) percent and (h) absolute number throughout the clinical course. Green designates to segmented neutrophils. Blue designates lymphocytes. Yellow designates monocytes. Red designates eosinophils. Purple designates basophils. Gray designates immature granulocytes.

The tumor was surgically resected and revealed atretic follicles with paracortical vascular penetration and onion-skinning, consistent with unicentric hyaline vascular type CD (HV-CD) (Figure 2(a)–2(c)). Notably, these histologic findings did not reveal morphologic evidence of the tumor arising from an accessory spleen and instead strongly favored lymph node involvement. Mature B cells and IgG were identified using pan-IgG immunohistochemical staining. Immunophenotyping of the biopsy and resected tumor samples supported a B-cell predominant lymphocyte population in the HV-CD tumor (Figure 2(d)). Taken together, the patient was diagnosed with HV-CD with cutaneous and pulmonary involvement.

Once the tumor was surgically removed, the patient appeared clinically improved but continued to struggle with dyspnea. Repeated pulmonary function tests revealed persistent severe obstruction consistent with bronchiolitis obliterans seen in the Castleman triad even after surgical resection of the tumor (Figure 2(e)). Over the disease course, the patient lost 16.8 kg, and prior to tumor removal, the patient had microcytic anemia and lymphopenia (Figure 2(f)–2(h)). In addition, he was at increased risk for bleeding with a prolonged prothrombin time (PT) of 14.6 s and high International Normalized Ratio (INR), C-reactive protein (CRP), and erythrocyte sedimentation rate (ESR) (PT 14.6 s, ref 11.8–13.8 s; INR 1.2, ref 0.9–1.1; CRP 26.5 mg/L ref <10 mg/L; ESR 13 mm/hr, ref <10 mm/hr). After the removal of the tumor in May 2018, complete blood counts with differentials revealed lymphopenia had resolved. Notably, numerous serum autoantibodies were positive from this patient both prior to and after removing the tumor (Table 1). Interestingly, a number of these autoantibodies remained positive in his serum more than a year after tumor removal. Due to the slow clinical improvement, persistent breathlessness, and positive autoantibodies, we elected to treat the patient with four infusions of rituximab. The rituximab provided little clinical benefit and was discontinued after the patient developed bilateral lung nodules, which were biopsied and diagnosed as acute fibrinous organizing pneumonia with poorly formed nonnecrotizing granulomatous inflammation. Since 2020, the patient has continued to have severe airway obstruction and is under evaluation as a potential lung transplant.

## 3. Discussion

Initially, we postulated the patient's autoantibodies may be cross-reactive with (retroperitoneal) tumor-associated antigens and would resolve after tumor removal. However, upon tumor removal, the presence of autoantibodies did not resolve and remained positive more than 1-year postoperation despite resolution of other associated clinical pathologies. The remaining circulating autoantibodies are reactive with autoantigens (anti-cyclic citrullinated peptide, anti-double-stranded DNA, and Sjogren's IgG autoantibodies) characteristic of diverse autoimmune diseases, suggesting a general loss of immunological tolerance may accompany CD.

Notably, it is becoming increasingly evident that certain inflammatory settings that lead to the production of proinflammatory mediators (e.g., IL-6, IL-1, TNF*α*) are associated with breaches in peripheral tolerance. As examples, Zika and coronavirus infections are typically accompanied by severe inflammation and autoantibody production that promote autoimmune syndromes and pathology, respectively [9, 10]. While the precise mechanism by which these infections appear to impair peripheral tolerance is not yet understood, proinflammatory cytokines have been shown to breach tolerance to grafts in both animal models and humans.

Indeed, CD has not only been associated with IL-6 production [11] but is also treated with tocilizumab [12]. In this scenario, CD tumor burden and associated IL-6 production break peripheral tolerance, which precipitated the patient's rheumatologic symptoms. There are anecdotal reports in the literature of pathogen-driven or sterile inflammatory settings that result in transient autoantibody production [3, 13]. Preexisting, autoreactive B cells found in healthy individuals could be released from peripheral restrain and contribute to the autoimmune pathophysiology observed and in presented in this report.

Typically, autoimmune responses are seen in multicentric CD and can present with PNP, autoimmune hemolytic anemia, interstitial lung disease, cytokine storm, and even mimic systemic lupus erythematosus (SLE) [1, 14, 15]. Autoimmune symptoms have been previously reported in UCD, yet these symptoms typically resolve after tumor removal [16–21]. Notably, UCD is proposed to be derived from follicular dendritic cells (FDCs), which specialize in antigen capture through immune complex formation [1]. The key functions of FDCs are to trap antigens, activate B cells, and promote follicular microarchitecture and formation of germinal centers [22]. In addition to these functions, FDCs have a unique capability of retaining native antigens in long-lasting antigen depots [22, 23]. This may explain why CD responds to rituximab and why immunomodulators are currently promising exploratory treatment modalities of CD over chemotherapy [24].

Importantly, both the tumor biopsy and resection showed a lymphocyte-predominant population. It is possible these relatively long-lived autoantibodies may reflect production by long-lived antibody-secreting plasma cells. Emerging evidence may suggest that long-lived antibody-secreting plasma cells are refractory to rituximab treatment [25–27] and may explain why our patient received minimal benefit from anti-CD20 therapy. This is the first report of a UCD patient with long-lived antibodies persisting in the serum more than a year after tumor removal. Our case suggests that the long-term clinical consequences of enduring autoantibodies in UCD are associated with lasting lung damage, potentially requiring future lung transplants. Importantly, these potential mechanisms appear to be taking place in a neoplastic and reactive lymph node. Interestingly, reactive lymph node involvement is frequently observed in a rare CD subvariant of TAFRO (thrombocytopenia, ascites/anasarca, myelofibrosis/fever, renal dysfunction/reticulin fibrosis, and organomegaly) [28]. A few studies have found unicentric HV-CD arising from an accessory spleen or reported Castleman tumors arising from other uncommon locations [29–33]. However, our case strongly favors the tumor arising from a lymph node.

The triad of problems in this patient with UCD have been previously reported, yet no large series has been reported [2–4, 21]. Our patient presented with PNP as the primary clinical complaint, accompanied by respiratory symptoms before/after CD excision. After treatment with various combinations of immunosuppressive and anti-inflammatory agents, most patients have great or total improvement in mucosal erosions, while their pulmonary function does not improve despite further immunosuppressive therapy. As with our patient, this sequalae of events has led to consider lung transplantation in such patients. In the patients reported so far, disease onset to lung transplant were 1, 2, and 5 years. All antibodies were negative or were present at low titers before the lung transplant. Altogether, this report identifies a case of HV-CD and coinciding autoantibody production with precipitating pemphigus vulgaris and bronchiolitis obliterans, which should be clinically recognized as the Castleman triad. CD patients presenting with autoimmune symptoms and autoreactive antibodies could have consequential long-term lung damage. While postsurgical rituximab offered minimal clinical benefit, earlier immunomodulatory intervention could be more effective to deplete B cells. Thus, autoantibody testing should be performed in CD patients and could potentially serve as an earlier interventional target. In this study, we highlight important and unexplored potential mechanisms that could be exploited to understand the pathophysiology, clinical course, and treatment of patients with CD.

## Figures and Tables

**Table 1:** Autoimmune and antibody testing.

Antibody	Suggestive for	Before tumor removal	After tumor removal
Anti-nuclear antibodies	Nonspecific	Positive	Negative
Anti-cyclic citrullinated peptide	RA	Positive	Positive
Anti-double-stranded DNA	SLE	Positive	Positive
Anticentromere	Scleroderma, CREST syndrome	Negative	–
Anti-ribosome P	SLE	Negative	–
ss-A/RO	SS, SLE, RA	Negative	–
ss-B/la	SS, SLE, RA	Negative	–
Anti-smooth muscle	Autoimmune hepatitis	Negative	–
Anti-protinase-3	Autoimmune vasculitis	Negative	–
Anti-myeloperoxidase	Autoimmune vasculitis	Negative	–
Rheumatoid factor	RA	Negative	–
Anti-ribonucleoprotein	SLE, MCTD	Negative	–
Anti-Scl70	Scleroderma	Negative	–
Anti-Jo1	Myositis	Negative	–
Sjogren's IgG	SS	Positive	Positive
*Mycoplasma pneumoniae* IgM	Acute infection	Positive	–
*Mycoplasma pneumoniae* IgG	Clearance/chronic infection	Positive	Positive
*Chlamydia pneumoniae* IgM	Acute infection	Negative	–
*Chlamydia pneumoniae* IgG	Clearance/chronic infection	Positive	Positive
HLA-B27	B27-associated diseases	Negative	–
PNP panel	Bullous pemphigoid/pemphigus vulgaris	Positive	–

RA, rheumatoid arthritis; SLE, systemic lupus erythematosus; SS, Sjogren's syndrome; MCTD, mixed connective tissue disease; PNP, paraneoplastic pemphigus.

## Data Availability

The authors declare that the data supporting the findings of this study are available within the paper. Any additional laboratory values, clinical data, or follow-up information can be made available from the corresponding author upon request.
